# Structural basis for recognition of Emi2 by Polo-like kinase 1 and development of peptidomimetics blocking oocyte maturation and fertilization

**DOI:** 10.1038/srep14626

**Published:** 2015-10-13

**Authors:** Jia-Lin Jia, Young-Hyun Han, Hak-Cheol Kim, Mija Ahn, Jeong-Woo Kwon, Yibo Luo, Pethaiah Gunasekaran, Soo-Jae Lee, Kyung S. Lee, Jeong Kyu Bang, Nam-Hyung Kim, Suk Namgoong

**Affiliations:** 1 Department of Animal Sciences, Chungbuk National University, Republic of Korea; 2 College of Pharmacy, Chungbuk National University, Republic of Korea; 3 Division of Magnetic Resonance, Korea Basic Science Institute, Ochang, Korea; 4 National Cancer Institute, National Institute of Health, Rockville, Maryland, United States

## Abstract

In a mammalian oocyte, completion of meiosis is suspended until fertilization by a sperm, and the cell cycle is arrested by a biochemical activity called cytostatic factor (CSF). Emi2 is one of the CSFs, and it maintains the protein level of maturation promoting factor (MPF) by inhibiting ubiquitin ligase anaphase promoting complex/cyclosome (APC/C). Degradation of Emi2 via ubiquitin-mediated proteolysis after fertilization requires phosphorylation by Polo-like kinase 1 (Plk1). Therefore, recognition and phosphorylation of Emi2 by Plk1 are crucial steps for cell cycle resumption, but the binding mode of Emi2 and Plk1 is poorly understood. Using biochemical assays and X-ray crystallography, we found that two phosphorylated threonines (Thr^152^ and Thr^176^) in Emi2 are each responsible for the recruitment of one Plk1 molecule by binding to its C-terminal polo box domain (PBD). We also found that meiotic maturation and meiosis resumption via parthenogenetic activation were impaired when Emi2 interaction with Plk1-PBD was blocked by a peptidomimetic called 103-8. Because of the inherent promiscuity of kinase inhibitors, our results suggest that targeting PBD of Plk1 may be an effective strategy for the development of novel and specific contraceptive agents that block oocyte maturation and/or fertilization.

Before birth, female gamete formation starts from immature oocytes, which are arrested in prophase and stored in primordial follicles until puberty. The cell cycle of oocytes is resumed after stimulation by sexual hormones. Subsequently, oocytes mature via germinal vesicle breakdown, asymmetric division, and polar body extrusion. Consequently, these mature metaphase II (MII) oocytes undergo ovulation. The cell cycle is then suspended to prevent parthenogenetic activation until fertilization by a sperm and is resumed by calcium-related signaling triggered by fertilization^1^.

The master regulator governing cell cycle control during oocyte maturation and fertilization is known as maturation-promoting factor (MPF)^2^, which is a heterodimer of cyclin B and cyclin-dependent protein kinase 1 (Cdk1)^3^,^4^. MPF activity increases in the course of oocyte maturation until metaphase I (MI). After the anaphase-telophase transition, mature MII oocytes maintain a high level of MPF activity, which arrests further progression of the cell cycle until fertilization. After fertilization, the high protein levels of MPF are decreased via degradation of cyclin B by ubiquitin-mediated proteolysis, which is promoted by ubiquitin ligase anaphase promoting complex/cyclosome (APC/C)^5^,^6^,^7^. “Cytostatic factor” (CSF) is a collective name of biochemical activities responsible for the process that prevents degradation of cyclin B; CSF serves to maintain the arrest of the cell cycle. Biochemical nature of CSF has been elusive for more than 30 years since the first identification of CSF in the 1970 s^2^, but its identity and molecular mechanisms have been elucidated significantly in the last decade.

One of CSFs, Emi2 (also known as F-box only protein 43) inhibits APC/C activity by binding to APC/C-cdc20; therefore, Emi2 blocks the ubiquitin-mediated proteolysis of MPF^8^,^9^,^10^. Usually, Emi2 expression starts at the beginning of the MII stage, and sharply decreases as a result of fertilization or oscillations in the calcium level^11^,^12^,^13^. Structural features of Emi2 are known: a destruction box (D-Box), a zinc-binding region (ZBR), and an RL-tail at the C terminus, which is capable of binding to APC/C-cdc20^14^. During the fertilization of an oocyte by a sperm, the elevated calcium concentration activates calmodulin-dependent protein kinase (CaMKII), which phosphorylates an N-terminal Ser/Thr of Emi2^8^. Subsequently, the phosphorylated threonines in Emi2 can be recognized by Plk1, which undergoes phosphorylation, and these phosphorylated sites serve as a recognition site for SCF, another class of ubiquitin ligases; SCF destabilizes Emi2 and activates APC/C^10^. Then, the activated APC/C can initiate degradation of cyclin B and downregulation of MPF; consequently, cell cycle progression can be resumed and meiosis II can be completed, as illustrated as Fig. 1A.

In *Xenopus* and in mice, the general scheme of calcium-mediated signal transduction has been well established, but the detailed molecular mechanism has been elusive. For instance, Plk1 is involved in Emi2 phosphorylation and destabilization^8^,^10^, but the mechanism of binding of Plk1 to Emi2 has not yet been determined. Plk1 is known to be involved in various cell cycle-related processes^15^, and recently it received attention as a target of anticancer treatments^16^. Although there are studies involving the kinase inhibitor BI2536^17^, which impairs mouse oocyte maturation^18^,^19^ and embryonic development of zebrafish embryos^20^, the function of Plk1 in oocyte maturation or fertilization in mammalian has not yet been clearly determined.

In this study, one of our aim was to elucidate recognition of Emi2 by the Polo-box domain (PBD) of Plk1 using X-ray crystallography and biochemical characterization. According to the structure of the complex of PBD with Emi2, we synthesized peptidomimetics blocking the interaction between PBD and Emi2. In addition, we tested whether each peptidomimetic can act as an antagonist of oocyte maturation or fertilization.

## Results

### Two putative binding sites for Plk1-PBD in the N terminus of Emi2 can bind two Plk1-PBDs simultaneously

**Figure 1:**
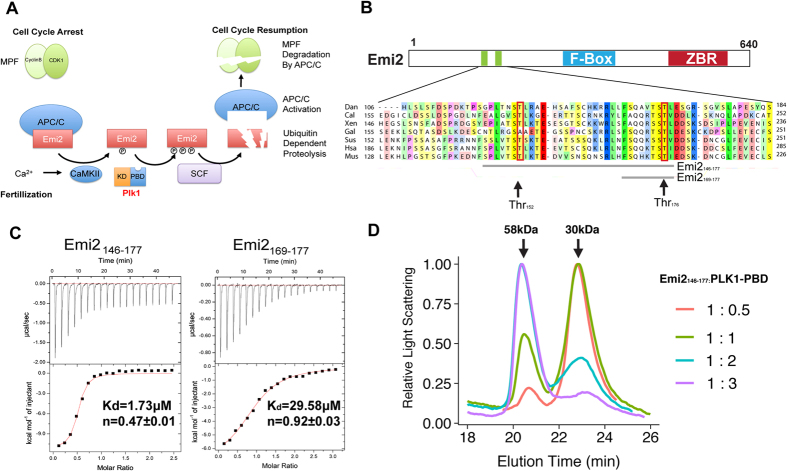
The presence of two Plk1-binding regions at the N terminus of mouse Emi2. (**A**) Diagram illustrating molecular events for cell cycle resumption after fertilization. In matured mammalian oocytes, maturation promoting factor (MPF) activity maintain high, because of ubiquitin ligase APC/C is inhibited by Emi2. After fertilization, increased Ca^2+^ activates calmodulin-dependent protein kinase II (CaMKII) and it phosphorylates N-terminal of Emi2. Plk1 is recruited by recognition of phosphothreonine residues in Emi2 by polo box domain (PBD) in Plk1 and subsequently phosphorylated Emi2 and phosphorylated Emi2 become substrate of another class of ubiquitin ligase, SCF and degradated by ubiquitin-proteasome. Activated APC-C can decrease MPF levels, therefore cell cycle for meiosis can be resumed. (**B**) Domain architecture of Emi2 and multiple-sequence alignment of Emi2 protein sequences from zebrafish (*Danio rerio*: Dan), Austrian ghostshark (*Callorhinchus milii*: Cal), *Xenopus* (*Xenopus laevis*: Xen), chicken (*Gallus*: Gal), *Homo sapiens* (hsa), pig (*Sus scrofa*: Sus), and mouse (*Mus musculus:* Mus). ZBR : Zinc Binding Region, Peptides containing a phosphorylated threonine (Emi2^146–177^ or Emi2^169–177^) are indicated with gray bars. Threonine residues that are subject to phosphorylation are indicated with a red box. (**C**) Binding affinity and binding stoichiometry of phosphorylated Emi2 peptides in relation to Plk1 Polo box domain (PBD). Isothermal titration calorimetry (ITC) with Emi2 peptides and Plk1-PBD was carried out as described in *Materials and Methods*. The dissociation constant (K_d_) and the stoichiometric ratio of an Emi2 peptide to Plk1-PBD (n) is also shown. (**D**) Size exclusion chromatography coupled with multiangle light scattering (SEC-MALS) of PLK1-PBD·Emi2^146–177^ complexes. Plk1-PBD and the phosphorylated Emi2^146–177^ peptide were mixed at various stoichiometric ratios (molar ratio of Plk1-PBD1 to Emi2^146–177^: 1:1, 1:2, and 1:3, respectively) and were separated by size exclusion chromatography. The measured molecular weight of each peak is indicated with arrows.

It is well known that calcium-dependent degradation of Emi2 and inactivation of MPF after fertilization are initiated by CaMK II-dependent phosphorylation of Emi2^8^,^10^,^21^. Subsequent recognition and phosphorylation by PLK1 results in formation of a signal for degradation of Emi2 by a SCF family ubiquitin ligase^21^ (Fig. 1A).

It has been shown that in *Xenopus* oocytes, phospho-Thr^170^ and phospho-Thr^195^ serve as recognition sites for PBD of *Xenopus* Plx1 (homolog of Plk1)^10^. In the case of mammalian Plk1 (including murine Plk1), the exact recognition site for Plk1-PBD in Emi2 is not known although a mutation of Thr^176^ in mouse Emi2, which corresponds to Thr^195^ in *Xenopus* Emi2, prevents degradation of Emi2 after fertilization^14^. We analyzed the N-terminal sequence alignment of Emi2 from various organisms including humans, mice, and *Xenopus* to search for possible recognition sites for Plk1-PBD (Fig. 1B). It was reported that Thr^195^, which is located in ^192^RSST^195^ motifs of *Xenopus* (corresponds to the ^174^KTST^177^ motif of murine Emi2), is phosphorylated by CaMKII and recognized by Plk1-PBD^10^. In addition to the “RSST” motif, there is a motif (corresponds to ^146^PLVTSTI^153^ in mouse Emi2) that is similar to the consensus motif of Plk1-PBD^22^,^23^, but its relevance to Plk1 recognition has not been established.

We synthesized two phosphothreonine-containing peptides spanning the amino acid positions 146–177 and 169–177 of Emi2, where two threonines (Thr^152^ and Thr^176^) were phosphorylated, and their binding affinity for Plk1-PBD was measured by isothermal titration calorimetry (ITC). As shown in Fig. 1C, the peptide Emi2^146–177^, which contains phospho-Thr^152^ and phospho-Thr^176^, showed higher binding affinity (K_d _= 1.73 μM) than did the peptide 169–177 (which has a single phosphothreonine: Thr^176^), which had a K_d_ value of 29.58 μM. ITC results suggested that the phosphopeptide 146–177 can bind to two Plk1-PBDs simultaneously (n = 0.47, expressed as a ratio of Emi2^146–177^ to Plk1-PBD), whereas phosphopeptide 169–177 binds an equimolar amount of Plk1-PBD (n = 0.92, expressed as a ratio of Emi2^169–177^ to Plk1-PBD). To confirm the simultaneous binding of PLK1-PBD to two putative binding sites of the PBD in Emi2, we mixed Plk1-PBD with Emi2^146–177^ at different stoichiometric ratios (1:0.5, 1:1, 1:2, or 1:3, respectively) and measured their molecular weight in the native state by size-exclusion chromatography coupled with multi angle light scattering (SEC-MALS).

As shown in Fig. 1D, the molecular weight of the Plk1-PBD-based complex is ~30 kDa, when the ratio of Plk1-PBD to Emi2^146–177^ is 1:0.5. The subsequent increase in the ratios of Plk1-PBD to Emi2^146–177^ (1:1 and 2:1), resulted in a dramatic shift in the molecular weight (from 30 to 58 kDa). When a threefold excess of Plk1-PBD was mixed with Emi2^146–177^, most of Plk1 was eluted in peaks corresponding to 60 kDa. These results show that both phosphothreonine-containing motifs near Thr^152^ or Thr^176^ can recruit Plk1-PBD.

### Structural analysis of the Plk1-PBD·Emi2 complex revealed Emi2′s unique binding modes

**Figure 2:**
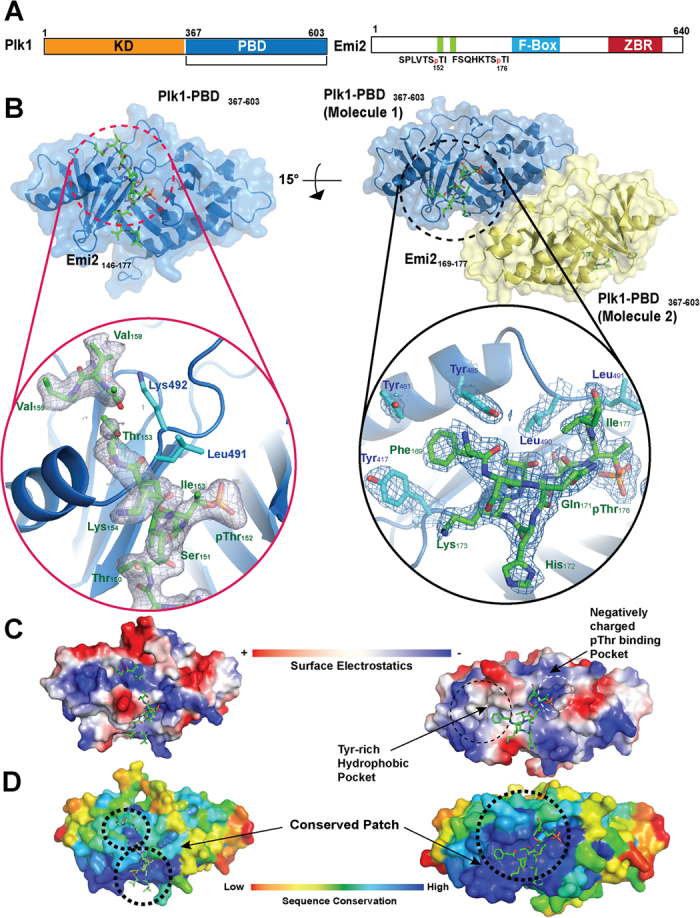
Crystal structure of Plk1 Polo box domain (PBD) and phosphorylated peptides derived from Emi2. (**A**) Domain organization of Plk1 and Emi2. Left: location of the kinase domain (KD) and the polo box domain (PBD) in Plk1 is indicated. Right: the location of the PBD-interacting part of Emi2 is highlighted in green. The phosphorylated threonine at amino acid positions 152 or 176 in Emi2 are also presented. ZBR: zinc-binding region. (**B**) Crystal structure of Plk1-PBD·Emi2^146–177^ (left) and Plk1-PBD·Emi2^169–177^. Plk1-PBD (blue) is shown as a cartoon Fig. and Emi2 fragments are shown as green sticks. Note that only electron density corresponding to amino acid residues 148–154 in Emi2^146–177^ is visible and was modeled. (**C**) Electrostatic surface representation (blue: positively charged, red: negatively charged) of Plk1-PBD bound to an Emi2-derived phosphopeptide. Left: Plk1-PBD·Emi2^146–177^, right: Plk1-PBD·Emi2^169–177^. (**D**) The surface of Plk1-PBD is colored according to residue conservation (conservation decreases from blue to red). Note that the Emi2-peptide binding regions including tyrosine-rich hydrophobic pockets and negatively charged regions binding Emi2^156–169^ are conserved (colored blue).

To elucidate the detailed molecular mechanism of recognition of Emi2 by Plk1-PBD, we determined structure of the complexes of PBD domain (367–603 of Plk1) with peptides Emi2^146–177^ or Emi2^169–177^ (Fig. 2A). First, we crystallized Plk1-PBD with Emi2^146–177^, which has two phosphothreonine residues (Thr^152^ and Thr^176^) and solved the complex’s structure at 2.4 Å (Fig. 2B, Left and supplementary movie 1). Although ITC and SEC-MALS indicated that the phosphorylated Emi2^146–177^ peptides can bind two PBDs simultaneously, only one binding event between the PBD and a phosphopeptide observed in an asymmetric unit in the crystal structure.

We examined the structure of the Plk1-PBD·Emi2^146–177^ complex containing various complexes of Plk1-PBD with cdc25 originated peptide or chemical 4j(Fig. S1), and we found that Emi2′s peptide-binding characteristics in relation to Plk1-PBD are similar to those previously reported for phosphopeptides (especially closely related to the Plk1-cdc25c structure)^24^. One considerable difference with the previous findings in the complex of Plk1-PBD with Emi2^146–177^ is that the extra electron density corresponds to Asp^157^, Val^158^, and Val^159^ bound to a negatively charged surface patch of Plk1-PBD (Fig. 2B, left in circle).

Because we could not identify the binding mode of phospho-Thr^176^ in the structure of the complex of PBD with Emi2^146–177^, we crystallized a shorter phosphopeptide (Emi2^169–177^, containing a single phospho-Thr^176^) with PBD and solved the crystal structure of this complex at 1.8 Å. (Fig. 2B, Right and Supplementary movie 1) In the asymmetric unit of the crystal structure, two molecules of Plk1-PBD bound with the one Emi2 peptide, respectively were found molecule 1 (shown in blue in Fig. 2D) displayed the whole sequence of amino acids (169 to 177) corresponding to Emi2^169–177^, while molecule 2 appeared as ^172^HKTSpTI^177^ (Fig. S2) of Emi2.

After careful analysis of the structure of this complex (Fig. 2B, Right), it is clear that the phosphothreonine residue (Thr^176^) binds to the canonical phospho-Thr/Ser-binding pocket of PBD; this mechanism is very similar to the known structures of binding complexes like PBIP1·PBD^25^ or cdc25C·PBD^24^.

Phe^169^ was found to be located in hydrophobic pockets, which are characterized by the presence of three tyrosine residues (Tyr^417^, Tyr^481^, and Tyr^485^). This binding behavior is different from that of peptides from native proteins including cdc25C·PBD^24^ or PBIP1·PBD^25^. As far as we know, ours is the first report of natural peptides occupying these hydrophobic pockets in Plk1-PBD.

To elucidate characteristics of the possible protein-protein interaction site in Plk1-PBD, we mapped sequence conservation scores calculated from the multiple-sequence alignment of various Plk1 homologs onto the surface of Plk1 (Fig. 2D and Supplementary movie 1). The two interacting binding pockets, such as the phospho-binding pocket and tyrosine-rich hydrophobic pockets, are located in the middle of an evolutionarily conserved region as shown in the structure of the complex in Fig. 2D. In addition, the negatively charged patch interacts with Asp^157^, Val^158^, and Val^159^ of Emi2, which also lie in the conserved region. It is obvious that this protein-protein interaction site is often characterized or predicted by means of the evolutionarily conserved surface patch^26^,^27^.

This evolutionary conservation of the phosphothreonine-binding pocket, hydrophobic pockets, and the negatively charged patch in PBD implies that these regions may be important for various protein-protein interactions and are probably utilized in Plk1-PBD interactions with other proteins. Collectively, these results suggested that the binding surface of PBD could accommodate various binding partners via subtle conformational changes after the binding of an interaction partner.

**Figure 3:**
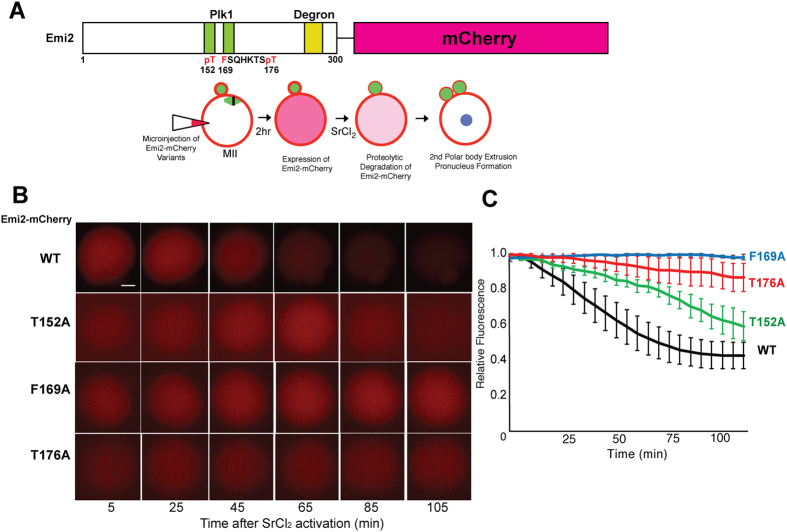
Testing the interactions of Emi2 with Plk1 based on Emi2 degradation after pathernogenetic activation of oocyte. (**A**)Scheme of Emi2^1–300^-mCherry reporter constructs used in the study. It contains Plk1 binding sites and degron required by recognition of SCF-mediated ubiquitinylation. Thr^152^, Phe^169^ and Thr^176^ mutated as alanine are indicated as red. After *in vitro* transcriptions, cRNAs were injected into MII oocyte and subjected to SrCl_2_-mediated parthenogenetic activation and decrease of fluorescence from mCherry was monitored by time-lapse imaging of oocytes. (**B**) Time-lapse microscopy of parthenogenetically activated oocyte injected with Emi2^1–300^-mCherry cRNAs. WT: wild type Emi2^1–300^-mCherry cRNAs; T152A: site-directed mutant Emi2^1–300^-mCherry containing Threonine^152^ to Alanine substitution; F169A: Phenylalanine^169^ to Alanine substitution; T176A: Threone^176^ to Alanine substitution; Representative figures of each groups corresponding 5, 25, 45, 65, 85 and 105 min after SrCl_2_ treatment were shown. (**C**) Quantification of fluorescence from oocyte injected with Emi2^1–300^-mCherry cRNAs. Normalized fluorescence from oocytes injected with wild type (Black, n = 7), T152A (Green, n = 6), F169A (Blue, n = 4) and T176A (Red, n = 5) at each time points (5 min interval) were measured and average of each time points were plotted. Error bars indicate standard error of mean (S.E.M).

To confirm Emi2 binding mode revealed from crystal structures, we generated series of point mutants on Emi2 gene, specifically on Thr^152^, Phe^169^ or Thr^176^ in Emi2^1–300^-mCherry reporter plasmids, which encode Plk1 recognition site and a ubiquitin-related degron^14^, then *in vitro* transcripts are subjected to inject on matured MII state oocyte and subjected to SrCl_2_-mediated pathernogenetic activation^28^, which induce rapid degradation of Emi2^9^,^14^ (Fig. 3A). If mutations on Plk1-binding residues on Emi2 impair interaction with Plk1, degradation of Emi2 would be affected. While fluorescence signal from wild type Emi2^1–300^-mCherry was rapidly decreased after pathernogenetic activation, fluorescence decays in all three mutants (Thr152Ala, Phe169Ala or Thr176Ala) are delayed compared with control (Fig. 3B and C). Especially, fluorescence decay of Phe169Ala mutant is minimal compared with two other mutants and wild type Emi2-mCherry, indicating that Phe^169^ in Emi2 is important for degradation of Emi2 after pathernogenetic activation. It also showed that both phosphor-threonine sites (152 and 176, respectively) in Emi2 contributed for recognition by Plk1.

### Peptidomimetic inhibitors targeting PBD can block maturation of mouse oocytes

**Figure 4:**
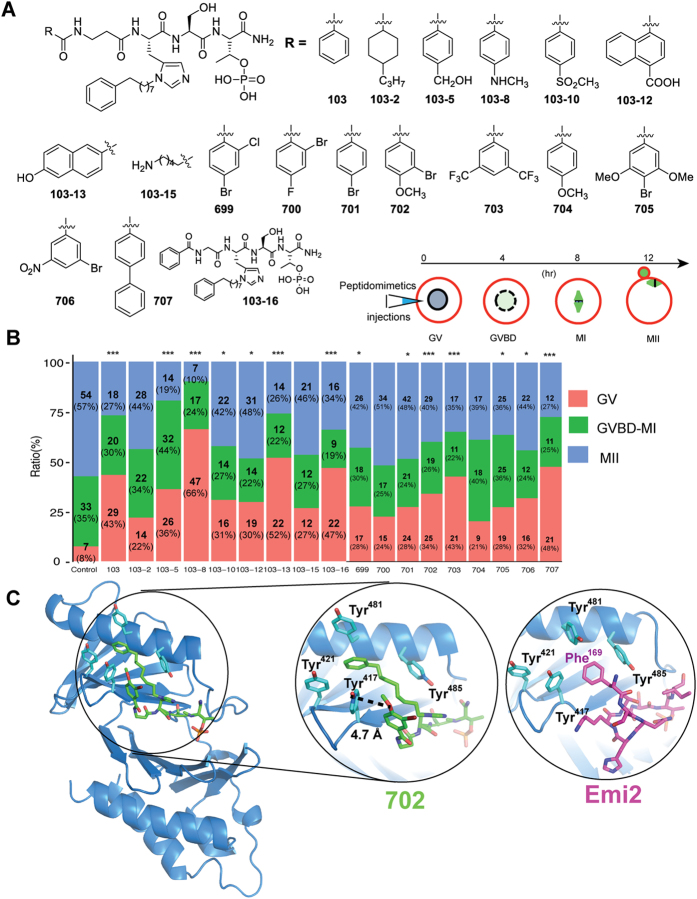
Peptidomimetics emulating the Plk1-PBD·Emi2 complex inhibit maturation of mouse oocytes. (**A**) Peptidomimetics resemble Plk1-PBD**·**Emi2 and bind to the Polo box domain (PBD) of Plk1. The compounds 103, 103-2, 103-5, 103-8, 103-10, 103-12, 103-13, 103-15 and 103-16 have been described previously^34^, and 699, 700, 701, 702, 703, 704, 705, 706 and 707 were characterized in this study. (**B**) Assessment of inhibitory activity of peptidomimetics on *in vitro* maturation (IVM) of mouse oocytes. Peptidomimetics were microinjected into immature mouse oocytes, which were subjected to IVM. GV: germinal vesicle, GVBD: germinal vesicle breakdown, MI: metaphase I, MII: metaphase II. Time (hr) required for maturation of an oocyte is indicated at the top of an arrow. Maturation status of mouse oocytes 12 hr after injection of a peptidomimetic was evaluated. At least three independent experiments were carried out and the ratios of oocytes at each developmental stage to all oocytes were plotted. Significant differences between the treatment groups and the control group are indicated by asterisk (***P ≤ 0.001, **P ≤ 0.01, *P ≤ 0.05). (**C**) Crystal structure of the Plk1-PBD·702 complex. PBD domain is shown in blue and peptidomimetic 702 is shown in green. In the enlarged circle, the structures of Emi2^169–177^ (violet) and peptidomimetic 702 (Green) are superimposed. Note that occupation of tyrosine-rich pockets by an alkylated imidazole ring (702) or a phenylalanine ring (Emi2^169–177^).

Interaction of Plk 1 and Emi2 is essential for fertilization^9^,^10^,^14^; depletion or inhibition of Plk1 impedes oocyte maturation^29^,^30^,^31^,^32^,^33^. Hence, we hypothesized that chemical inhibition that disrupts the specific interaction of Plk1 and Emi2 may be an attractive strategy for the development of novel contraceptive agents. We observed a high degree of similarity between the binding modes of PBD with Emi2 and PBD with PBIP1; therefore, first, we tested the effect of peptidomimetics (shown in Fig. 4A) that were previously described as 103 series^34^ in experiments with mouse oocyte maturation by microinjections of peptidomimetics.

Furthermore, new peptidomimetics (699, 700, 701, 702, 703, 704, 705, and 706) that we developed according to the crystal structure of the complex of Plk1- PBD with Emi2, were introduced by microinjection into immature mouse oocytes, and the resulting *in vitro* maturation was analyzed. The small molecule bg-34^35^ without phosphor-threonine, therefore has no binding affinity for Plk1-PBD was used as a negative control. As shown in Fig. 4B, most of the peptidomimetics caused a significant decrease in oocyte maturation rates, while with the negative control (bg34) only 8% of oocytes stayed at the GV stage.

Among the tested peptidomimetics, 103-8 (which contains a *p*-amino methyl group in the phenyl ring), showed the strongest inhibitory properties: 66% of oocytes were arrested at the GV stage and only 10% of oocytes reached the MII stage. Previously, it was shown that the 103-8 derivative also has strong binding affinity for Plk1-PBD (*in vitro* activity half-maximal inhibitory concentration [IC_50_] 40 nM)^34^.

To identify the binding modes responsible for this strong activity, we tried to solve the structure of the complex of 103-8 with Plk1-PBD. Unfortunately, our attempts to crystallize the complex of 103-8 with Plk1-PBD were not successful. Instead, we obtained structure of the complex of PBD with peptidomimetic 702, which has a phenyl substituent that anchors bromide and methoxy groups at *meta*- and *para*-positions, respectively. As shown in Fig. 4C, the structure of the complex of Plk1-PBD with 702 is very similar to the previously solved structure of Plk1-PBD with 103-5; the latter peptidomimetic contains a hydroxy methyl group. The distance between Tyr^417^ and the oxygen atom of the methoxy group in 702 was found to be 4.7 Å. This finding could be attributed to the orientation of 702′s functional groups toward Tyr^417^. The subsequent hydrogen bonding between Tyr^417^ and the oxygen of the methoxy group in 702 determines the strong binding affinity.

### Injection of the peptidomimetic 103-8 inhibits formation of meiotic spindles and delays parthenogenetic activation

**Figure 5:**
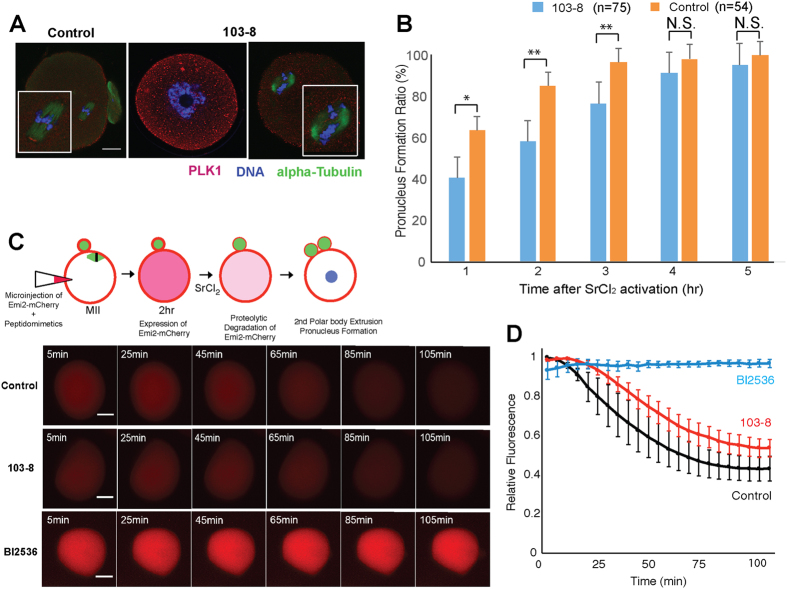
The peptidomimetic 103-8 impairs oocyte maturation and delays parthenogenetic activation of mouse oocytes. (**A**) Effects of 103-8 injections on oocyte maturation. Control (oocytes injected with unphosphorylated BG34) and 103-8-injected oocytes were stained for PLK1 (red), α-tubulin (green), and DNA (blue). Bar = 20 μm. **(B)** Effects of 103-8 injections on parthenogenetic activation of mouse oocytes. Mature metaphase II (MII) oocytes were microinjected with a control compound (orange) or 103-8 (blue), and subjected to parthenogenetic activation by SrCl_2_. The progression of parthenogenetic activation was assessed by the formation of a pronucleus (examination under an inverted microscope). Statistical significance was tested by chi-square test (**P ≤ 0.01, *P ≤ 0.05, and N.S. P > 0.05). (**C**) Time-lapse microscopy of parthenogenetically activated oocyte injected with Emi2^1–300^-mCherry cRNAs. Control: wild type Emi2^1–300^-mCherry cRNAs injected with control BG34 control; 103-8: Emi2^1–300^-mCherry cRNAs injected with 103-8 peptidomimetic; BI2536: Emi2^1–300^-mCherry cRNAs injected and subjected to SrCl_2_ treatment in the presence of 100 nM BI2536. Bar = 40 μm. (D) Normalized fluorescence from oocytes injected with control (Black, n = 9), 103-8 (Red, n = 12) and BI2536 (Blue, n = 3) at each time points (5 min interval) were measured and average of each time points were plotted. Error bars indicate standard error of mean (S.E.M).

Next, we characterized the phenotypes caused by injection of the peptidomimetic 103-8 into mouse oocytes. As shown in Fig. 4B, more than 60% of oocytes failed to continue the cell cycle and stayed at the GV stage. Those oocytes that proceeded to the MI stage often showed abnormalities in the spindle formation, as shown in Fig. 5A. It was reported that Plk1 is localized in the microtubule organizing center at the GV breakdown stage of oocytes and in spindle poles at the MI stage^32^,^36^,^37^. We obtained similar results when the negative control (bg-34) was injected into oocytes, as shown in Fig. 5A. In contrast, injection of 103-8 disrupted localization of Plk1 in spindle poles and caused abnormal spindle morphology, indicating that localization of Plk1 in the meiotic spindle can be inhibited by PBD antagonists.

Next, we tested whether treatment of mature oocytes (at MII) with a peptidomimetic prevents parthenogenetic activation caused by treatment with SrCl_2_. As shown in Fig. 5B, oocytes that were injected with 103-8 showed delayed appearance of the pronucleus after activation by SrCl_2_, indicating that injection of 103-8 into an oocyte effectively delayed the resumption of meiosis. This effect is probably mediated by Plk1 inhibition and therefore by blockade of Emi2 degradation. To confirm directly whether injection of a peptidomimetic can block or delay Emi2 degradation after parthenogenetic activation, we injected each of Emi2^1–300^-mCherry reporter cRNAs (which encode Plk1 recognition sites and a ubiquitin-related degron^14^), into MII oocytes along with either 103-8 or control. Then, the oocytes were subjected to activation by SrCl_2_ and were monitored for possible disappearance of the mCherry fluorescent signal (by time lapse microscopy) after the activation.

As shown in Fig. 5C,D, when the negative control (bg34) was injected along with an Emi2-mCherry cRNA, the subsequent addition of SrCl_2_ reduced the red fluorescence rapidly, indicating that Emi2 degradation took place after the parthenogenetic activation. In contrast, addition of a Plk1 kinase domain inhibitor (BI2536, 100 nM) did not cause any reduction in fluorescence even after 100 min of parthenogenetic activation by SrCl_2_. These results revealed that chemical inhibition of Plk1 could prevent degradation of Emi2-mCherry, by blocking the parthenogenetic activation.

Meanwhile, injection of 103-8 caused a delay in the fluorescence decrease in comparison with the negative control after the activation. These observations revealed that 103-8 partially blocked parthenogenetic activation, just as in the pronucleus formation assay (Fig. 5D). These results suggested that activity of Plk1 can be targeted by inhibitors in both domains — the catalytic domain (by BI2536) and the PBD (by 103-8) — and this approach could inhibit the fertilization process in a mammalian oocyte.

## Discussion

Here, we studied the structure of a complex of Plk1-PBD with phosphorylated peptides derived from Emi2. On the basis of this structure, we designed the peptidomimetics and examined their ability to block oocyte maturation or parthenogenetic activation.

We found two putative Plk1-PBD-binding regions in Emi2, which can mediate formation of a complex with two Plk1-PBD molecules (one each) simultaneously *in vitro*. Other *in vitro* studies, such as those involving *Xenopus* egg extracts, showed that phosphorylation on Thr^170^ and Thr^195^ (correspond to Thr^152^ and Thr^176^ in murine Emi2) is necessary for degradation of Emi2^10^,^38^; however, the effects of these phosphothreonines on the Plk1-binding ability or their functions in mammalian Emi2 regulatory mechanisms have not been known to date. Our experiments with phosphorylated Emi2 peptides showed that phosphorylation on both threonine residues in the Emi2 fragments can recruit two PBDs simultaneously, with a significant increase in binding affinity. One possible explanation is that simultaneous binding of two Plk1-PBDs via Thr^170^ and Thr^195^ may facilitate multiple phosphorylation of Plk1-PBD in complex with Emi2. Because two degradation motifs of Emi2 are prone to phosphorylation by Plk1 and to recognition by a ubiquitin ligase of the SCF family^38^,^39^,^40^, simultaneous binding and recruitment of two Plk1-PBDs to Emi2 may facilitate Plk1-mediated degradation of Emi2. Nonetheless, the exact physiological significance of the two possible Plk1-PBD-binding sites for Emi2 degradation needs further research.

The structure of the complex of Plk1-PBD with Emi2 confirmed that the Emi2-binding regions are generally similar to those in known structures, but there are several unique features. For example, Phe^169^ of Emi2 is located in the middle of a Tyr-rich hydrophobic pocket; this seems to be the first report that a natural peptide binds to Tyr-rich hydrophobic pocket. This result points to importance of the Tyr-rich hydrophobic pocket for recognition of Emi2; this importance was also shown for peptidomimetic 4j, in which the long-chain alkyl phenyl on histidine binds to the Tyr-rich channel and increases the binding affinity for Plk1-PBD more than 1000-fold in comparison with the parent peptide^41^.

We explored the biological effects on mammalian oocyte maturation or fertilization for the peptidomimetics blocking interaction between the PBD and Emi2. It has been reported that inhibition of Plk1 by antibody injection^32^,^37^,^42^ or a kinase inhibitor^19^ impairs oocyte maturation, but there are no studies showing that targeting of the PBD of Plk1 can suppress meiosis resumption, which is mediated by calcium signaling and degradation of Emi2. In the present study, we found that PBD-targeting peptidomimetics suppress *in vitro* mouse oocyte maturation effectively. The results in this study suggest the possibility that targeting of Plk1 by PBD-binding antagonists may inhibit oocyte maturation, although more detailed research should be required to evaluate its effectiveness as potential target for contraceptive agent development. Furthermore, we also confirmed prevention of resumption of meiosis by inhibition of Plk1 by means of a kinase domain inhibitor (BI2536)^17^,^43^, which blocks Emi2 degradation after parthenogenetic activation by SrCl_2_, indicating the possibility that Plk1 could be a target for development for contraceptive agent.

Meanwhile, the peptidomimetic 103-8 targeting the PDB also delayed the parthenogenesis although its effect was not so dramatic as that of BI2536. On the other hand, due to the high degree of similarity among kinase domains’ ATP-binding pockets, achieving good specificity is a difficult task. In addition with other kinases, BI2536 have been reported to bind and inhibit bromodomain BRD4 at nanomolar concentration^44^. Because PBD exists only in the Plk family (Plk1 through Plk4), targeting of this site can result in high specificity along with enhanced binding affinity; these properties may facilitate the development of new drugs.

Altogether, our results indicate that PBD-targeting inhibitors could be potential candidates for prevention of mammalian oocyte maturation or fertilization. Furthermore, we showed that an *in vitro* maturation system that is based on mammalian oocytes or the Emi2-mCherry reporter during parthenogenetic activation may be used as alternative cell-based systems for screening of Plk1 inhibitors. Considering the importance of Plk1 as a target of anticancer drugs, such an alternative cell-based assay should be useful for the development of a next generation of Plk1-inhibitory agents.

## Methods

### Creation of the expression construct and expression and purification of Plk1-PBD

Mouse Plk1-PBD (amino acid residues 367–603) was expressed as a recombinant protein containing an N-terminal His_6_ tag in the pRSF-Duet vector (BD Bioscience). A TEV protease cleavage site (sequences ENLYFQ/S, where the amino acid residue between Q and S is cleaved) was engineered between the His_6_ tag and PBD. The protein was expressed in *Escherichia coli* BL21 (DE3) at 20 °C; the expression was induced by 0.4 mM isopropyl β-D-1-thiogalactopyranoside (IPTG) and the bacterial cells were cultured for 12 hr. The cells were harvested and resuspended in buffer A (25 mM HEPES pH 7.5, 5 mM β-mercaptoethanol, 300 mM NaCl, 10 mM imidazole, and 0.1 mM phenylmethanesulfonylfluoride [PMSF]) and purified by Ni-NTA affinity chromatography (Qiagen). The eluted His-tagged PBD domain was digested with TEV protease (1:100 molar ratio) overnight, with dialysis against buffer A. Undigested protein was removed by His tag chromatography. The concentrated proteins were further purified on the HiLoad 16/60 Superdex 75 gel filtration column equilibrated with the buffer consisting of 25 mM HEPES pH 7.5, 5 mM β-mercaptoethanol, 300 mM NaCl, and 10% glycerol. The purified proteins were concentrated to 10 mg/mL, flash frozen in liquid nitrogen, and stored at −70 °C until use.

### Peptide synthesis

All peptides were prepared by Fmoc SPPS methods with the Rink amide resin and initial loading at 0.61 mmol/g unless stated otherwise. Fmoc-protected amino acids were purchased from Novabiochem. The resins were allowed to swell in *N*,*N*-dimethylformamide (DMF) for 45 min prior to the synthesis. For sequence extension, the Fmoc-protected amino acid (5.0equivalents) was activated by treatment with 1-*O*-benzotriazole-*N*,*N*,*N′*,*N′*-tetramethyluronium hexafluorophosphate (HBTU; 5.0 equiv.), 1-hydroxybenzotriazole (HOBt) (5.0 equiv.), and *N*,*N*-diisopropylethylamine (10.0 equiv) in DMF (2 mL) for 2 min. This solution was added to the free amine on the resin, and the coupling reaction was allowed to proceed for 1 hr with vortex stirring. After washing with DMF, we accomplished Fmoc deprotection by means of 20% piperidine in DMF (1 time for 10 min, 2 times for 3 min). The resin was washed once again, and the process was repeated for the next amino acid; finally the resin was washed with DMF, methanol, dichloromethane, and ether and then dried under vacuum. Linear peptides were cleaved from the resin with 5% triisopropylsilane (TIS) and 5% H_2_O in trifluoroacetic acid (TFA, approximately 2 mL of TFA per 100 mg of the resin) for 2 hr. The resulting mixture was added to cold ether to precipitate the peptide and then filtered. Reverse-phase high-performance liquid chromatography (RP-HPLC) was carried out on a preparative Vydac C_18_ column (15 μm, 20 mm × 250 mm), with an appropriate water/acetonitrile gradient in the presence of 0.05% TFA. The final purity of the peptides (>98%) was assessed by RP-HPLC on an analytical Vydac C_18_ column (4.6 mm × 250 mm, 300 Å, 5 μm particle size). Molecular weight of the purified peptides was determined by matrix-assisted laser desorption ionization time-of-flight mass spectrometry (MALDI-TOF MS; Shimadzu, Japan). A Fmoc-protected histidine derivative [*N*-(*π*)-PhC_8_H_18_-] was prepared according to our previously reported procedure^45^.

The following are characteristics of the peptides; peptide 103: MS (MALDI-TOF) m/z = 786.39 [M+H]^+^, 103-2: MS (MALDI-TOF) m/z = 834.50 [M+H]^+^, 103-5: MS (MALDI-TOF) m/z = 816.44 [M+H]^+^, 103-8: MS (MALDI-TOF) m/z = 815.43 [M+H]^+^, 103-10: MS (MALDI-TOF) m/z = 864.53 [M+H]^+^, 103-12: MS (MALDI-TOF) m/z = 880.49 [M+H]^+^, 103-13: MS (MALDI-TOF) m/z = 852.44 [M+H]^+^, 103-15: MS (MALDI-TOF) m/z = 795.52 [M+H]^+^, 103-16: MS (MALDI-TOF) m/z = 772.45 [M+H]^+^, 699: MS (MALDI-TOF) m/z = 899.04 [M+2H]^+^, 700: MS (MALDI-TOF) m/z = 882.97 [M+H]^+^, 701: MS (MALDI-TOF) m/z = 865.13 [M+2H]^+^, 702: MS (MALDI-TOF) m/z = 895.19 [M+2H]^+^, 703: MS (MALDI-TOF) m/z = 923.25 [M+2H]^+^, 704: MS (MALDI-TOF) m/z = 816.99 [M+H]^+^, 705: MS (MALDI-TOF) m/z = 925.14 [M+2H]^+^, 706: MS (MALDI-TOF) m/z = 910.20 [M+2H]^+^, 707: MS (MALDI-TOF) m/z = 863.47 [M+2H]^+^.

### Crystallization, data collection, and refinement of data on the complexes Emi2^169–177^·Plk1-PBD, Emi2^146–177^·Plk1-PBD, and peptidomimetic 702·Plk1-PBD

The complex Emi2^169–177^**·**Plk1-PBD (^169^FSQHKTSpTI^177^, where pT indicates phosphothreonine) was formed by mixing the phosphorylated Emi2^169–177^ peptide with Plk1-PBD at stoichiometry 2:1. The crystal was screened by the hanging-drop buffer diffusion method. The crystal was grown at 20 °C in 15% polyethylene glycol (PEG) 3350 and 0.1 M Tris-HCl (pH 8.5) and frozen in the buffer consisting of 30% glycerol, 15% PEG 3350, and 0.1 M Tris-HCl pH 8.5. In the case of the Emi2^146–177^**·**Plk1-PBD complex, the crystal was prepared in the buffer consisting of 10 mM MgCl_2_, 5 mM NiCl_2_,15% PEG 3350, and 0.1 M HEPES (pH 7.0) and cryoprotected by the addition of 20% of ethylene glycol. The complex of peptidomimetic 702 with Plk1-PBD was crystallized in the buffer consisting of 28% 2-propanol, 3% PEG 200, and 0.1 M Bis-Tris (pH 6.5) and frozen in 20% ethylene glycol. Datasets were recorded in Pohang Accelerator Laboratory 7A and processed by means of XDS^46^. Molecular replacement solutions were found by means of the complex with PLHSpTA phosphopeptide in PBIP1 and Plk1-PBD1 (Protein DataBank [PDB] ID: 3P2Z) as a search model by means of Phaser^47^. Refinement was performed in the PHENIX-Refine software^48^, with manual rebuilding by means of COOT^49^. Detailed data and refinement statistics are presented in Table 1.

### Isothermal titration calorimetry (ITC)

The calorimetric-titration analyses were performed on an Auto-iTC 200 titration system (Malvern, Malvern, UK). In a typical experiment, 2-μL aliquots of a phosphorylated peptide were injected by means of a 110-μL syringe into a rapidly mixed (500 rpm) solution of Plk1-PBD (cell volume 370 μL). Control experiments involved injection of identical amounts of the peptide solution into the buffer without Plk1-PBD. The concentration of Plk1-PBD was 0.1 mM, and that of the peptides was 1.2 mM; the concentrations were determined by amino acid analysis. Titration analyses were conducted at 15 °C in a buffer consisting of 20 mM Tris-Cl (pH 8.0), 300 mM NaCl, and 3 mM DTT. The isotherms, corrected for dilution/buffer effects were fitted in the Origin ITC Analysis software according to manufacturer’s protocols. The nonlinear least-square method was used to fit the titration data and to calculate the errors. In accordance with the structural data, 1:1 stoichiometry was assumed, and the data were fitted to a one-site binding model. From the binding curve, we extracted the values of enthalpy and binding affinity. Thermodynamic parameters were calculated using the formulas ∆G = −RT lnKa and ∆G = ∆H − T∆S.

### Size exclusion chromatography with multiangle light scattering (SEC-MALS)

We fractionated 63 μM PLK1-PBD mixed with the Emi2^146–177^ peptide at various stoichiometric ratios (0.5:1, 1:1, 2:1, or 3:1, respectively) and 100 μL of complexes by size exclusion chromatography on a WTC-015S5 column (Wyatt Technology) coupled to an Agilent 1100 HPLC system (Agilent Technologies). The molecular species that were separated by the column were analyzed by DAWN HELEOS II multi-angle light scattering (MALS) detector and an Optilab rEX refractive index detector; molecular weights of the molecular species were calculated in the Astra software (Wyatt Technology Corp.).

### Mouse Oocyte collection and the *in vitro* maturation procedure

All procedures with mice were conducted according to the Animal Research Committee guidelines of Chungbuk National University, Korea and all animal manipulations and experimental protocols were approved by the Animal Research Committee, Chungbuk National University, Korea (approval no. CB-R28).

The 4- to 6-week-old imprinting control region (ICR) mice were sacrificed by cervical dislocation at 48 hr after injected with 10 IU of pregnant mare’s serum gonadotropin (PMSG, Daesung Biochemical, Daejun, Korea) and germinal vesicle (GV) stage oocytes were collected from ovaries. Cumulus-free and intact-GV follicular oocytes were released from large antral follicles via puncture with a needle in the M2 medium (Sigma-Aldrich, St. Louis, MO, USA) with 60 μg/mL penicillin and 50 μg/mL streptomycin. All cell culture were maintained in the M16 medium (Sigma-Aldrich) at 37 °C in a humidified atmosphere containing 5% of CO_2_. Cumulus cell-enclosed metaphase II-arrested eggs were obtained from mice of the same strain. The cumulus cell masses surrounding the eggs were removed by brief exposure to 300 IU/mL hyaluronidase in the M2 medium.

### Preparation of cRNAs

pRN3-Emi2^1−300^-mCherry plasmids containing the Plk1 recognition site and a degron for a SCF family ubiquitin ligase were constructed from synthetic Emi2^1–300^ gene fragments and subcloned into the pRN3 vector as an mCherry fusion by means of Gibson Assembly^50^. The plasmids were linearized by digestion with *Sfi*I; then cRNA was synthesized with T3 mMessage mMachine (Life Technologies). *In vitro* transcripts were purified by phenol-chloroform extraction and isopropyl alcohol precipitation and stored at −80 °C until use.

### Chemical injection and parthenogenetic activation

GV stage oocytes were injected with various peptidomimetics at 300 μM in a 50% aqueous solution (w/v) of dimethyl sulfoxide. Peptidomimetics were injected into oocyte by means of a Eppendorf Femto Jet (Eppendorf AG, Hamburg, Germany) and a Nikon Diaphot ECLIPSE TE300 inverted microscope (Nikon UK Ltd., Kingston upon Thames, Surrey, UK) equipped with a Narishige MM0-202N hydraulic three-dimensional micromanipulator (Narishige Inc., Sea Cliff, NY, USA) and subjected to *in vitro* maturation as described above. As a control, the BG34 reagent with threonine residues instead of phosphothreonine^35^ was injected into oocytes at 300 μM. To test the oocytes for parthenogenetic activation, we collected mature MII oocytes and injected them with various reagents as described above for GV oocytes. Parthenogenetic activation was accomplished by incubating the MII oocytes in the calcium-free CZB medium^51^ supplemented with 5 mM SrCl_2_ at 5% CO_2_ (v/v, in humidified air) and 37 °C for 2 hr^52^.

### Immunostaining and confocal microscopy

To immunostain oocytes for Plk1 or alpha-tubulin, the cells were fixed in 4% paraformaldehyde in phosphate-buffered saline (PBS) and then transferred to a membrane permeabilization solution (0.5% Triton X-100) and kept there for 1 hr. After 1 hr in blocking buffer (PBS containing 1% bovine serum albumin), the oocytes were incubated overnight at 4 °C with an anti-human Plk1 antibody (Santa Cruz Biotech, Santa Cruz, CA, USA). After three washes with PBS containing 0.1% Tween 20 and 0.01% Triton X-100, the oocytes were incubated with an Alexa Fluor 488-conjugated goat anti-mouse IgG (1:100 dilution) for 1–2 h at room temperature. For staining of the cytoplasmic actin mesh or cortical actin, the oocytes were fixed and stained with phalloidin-tetramethylrhodamine (10 μg/mL), which labels F-actin. For staining with an anti-α-tubulin-fluorescein isothiocyanate antibody (1:200 dilution), the oocytes were incubated with this reagent for 1 h, washed three times in washing buffer for 2 min each time, incubated with Hoechst 33342 (10 μg/mL in PBS) for 15 min, and then washed three times in washing buffer. The samples were mounted onto glass slides and examined under a confocal laser scanning microscope (Zeiss LSM 710 META, Jena, Germany, with a 40× water immersion objective lens).

### Microinjection of cRNA and time lapse monitoring of parthenogenetic activation

Each cRNA was microinjected into *in vitro*-matured MII stage mouse oocytes as described previously^53^. To monitor downregulation of Emi2-mCherry during parthenogenetic activation, each Emi2-mCherry cRNA, which was diluted to 1 μg/μL, was microinjected into the cytoplasm of a fully-grown GV stage oocyte by means of an Eppendorf Femto Jet (Eppendorf AG, Hamburg, Germany), and the cells were examined under a Nikon Diaphot ECLIPSE TE300 inverted microscope (Nikon UK Ltd., Kingston upon Thames, Surrey, UK) equipped with a Narishige MM0-202N hydraulic three-dimensional micromanipulator (Narishige Inc., Sea Cliff, NY, USA). After the injection, the oocytes were incubated in the M16 medium for 2 hr, then subjected to parthenogenetic activation: the MII oocytes were incubated in the calcium-free CZB medium^51^ supplemented with 5 mM SrCl_2_ at 5% CO_2_ (v/v, in humidified air) and 37 °C.

### Data analysis

For each treatment, at least three replicates were performed. Statistical analyses were conducted using Welch’s t-test, Pearson’s chi-square test, Fisher’s exact test, or an analysis of variance (ANOVA), followed by Tukey’s multiple comparisons of means by R (R Development Core Team, Vienna, Austria). Data are expressed as mean ± standard error of the mean, and p < 0.05 was considered significant.

## Additional Information

**How to cite this article**: Jia, J.-L. *et al*. Structural basis for recognition of Emi2 by Polo-like kinase 1 and development of peptidomimetics blocking oocyte maturation and fertilization. *Sci. Rep*. **5**, 14626; doi: 10.1038/srep14626 (2015).

## Supplementary Material

Supplementary Information

Supplementary Movie 1

## Figures and Tables

**Table 1:** Crystallographic data and refinement statistics.

	Plk1-PBD·Emi2^146–177^	Plk1-PBDEmi2^169–177^	Plk1-PBD·702
*Diffraction data*			
Wavelength, Å	1.0	1.0	1.0
Space group	P 1 2 1	P 1 2_1_ 1	P 2_1_ 2_1_ 2_1_
Unit cell *a*/*b*/*c*, Å	35.6, 63.3, 56.8	56.8, 70.5, 59.2	57.1, 59.9, 67.0
Unit cell *α*/*β*/*γ*, °	90.0, 97.2, 90.0	90.0, 90.1, 90.0	90.0, 90.0, 90.0
Resolution, Å	2.5–28.4 (2.5–2.6)	1.9–35.4 (1.9–2.0)	2.3–44.7 (2.3–2.4)
Completeness, %	97.9 (89.3)	98.4 (91.1)	90.41(76.8)
Multiplicity	4.0(3.7)	7.4 (5.4)	3.6(2.6)
*R*_*merge*_^a^, %	18.8(43.8)	5.0 (18.1)	8.9(2.5)
*I/σ*	8.5(3.47)	21.7 (6.0)	23.4(3.9)
*Refinement*			
Resolution, Å	2.5–28.4	1.9–35.4	2.3–44.7
No of reflections	8266	36843	9156
Completeness, %	94.1	99.9	90.5
*R*_*factor*_^b^, %	21.8	19.8	19.6
*R*_*free*_^c^, %	28.8	24.4	27.7
*Rmsd* bonds, Å	0.013	0.007	0.014
*Rmsd* angles, °	2.115	1.103	1.812
*B-factor* protein, Å^2^	37.4	36.2	32.4
*B-factor* solvent, Å^2^	44.4	39.9	34.0
PDB accession code	5DMV	5DMS	5DMJ

Values in parentheses correspond to highest resolution shell.

^a^*R*_*merge *_= Σ_*hkl*_(*I* − <*I*>)/Σ*I*, where *I* and <*I*> are the observed and mean intensities of all observations of reflection *hkl*, including its symmetry-related equivalents.

^b^*R*_*factor *_= Σ_*hkl*_||*F*_*obs*_*|* − |*F*_*calc*_*|*|/Σ|*F*_*obs*_|, where *F*_*obs*_ and *F*_*calc*_ are the observed and calculated structure factors of reflection *hkl*.

^c^*R*_*free*_, *R*_*factor*_ calculated for a randomly selected subset of the reflections (5%) that were omitted during refinement.
