# Supplementary material for: Structural basis for recognition of Emi2 by Polo-like kinase 1 and development of peptidomimetics blocking oocyte maturation and fertilization
Source: Sci Rep. 2015 Oct 13;5:14626. doi: 10.1038/srep14626 (PMC4602232; doi:10.1038/srep14626)
Supplement: Supplementary Information [file srep14626-s1.pdf]

Supplementary Informations

Structural basis for recognition of Emi2 by Polo-like kinase 1 and the implications for development of peptidomimetics blocking oocyte maturation and fertilization

Jia-Lin Jia [1+], Young-Hyun Han[1+], Hak-Cheol Kim[1+], Mija Ahn[3], Jeong-Woo Kwon[1], Yibo Luo[1], Pethaiah Gunasekaran[1], Soo-Jae Lee[2], Kyung S. Lee[4], Jeong Kyu Bang[3*], Nam-Hyung Kim[1*], and Suk Namgoong[1*]*

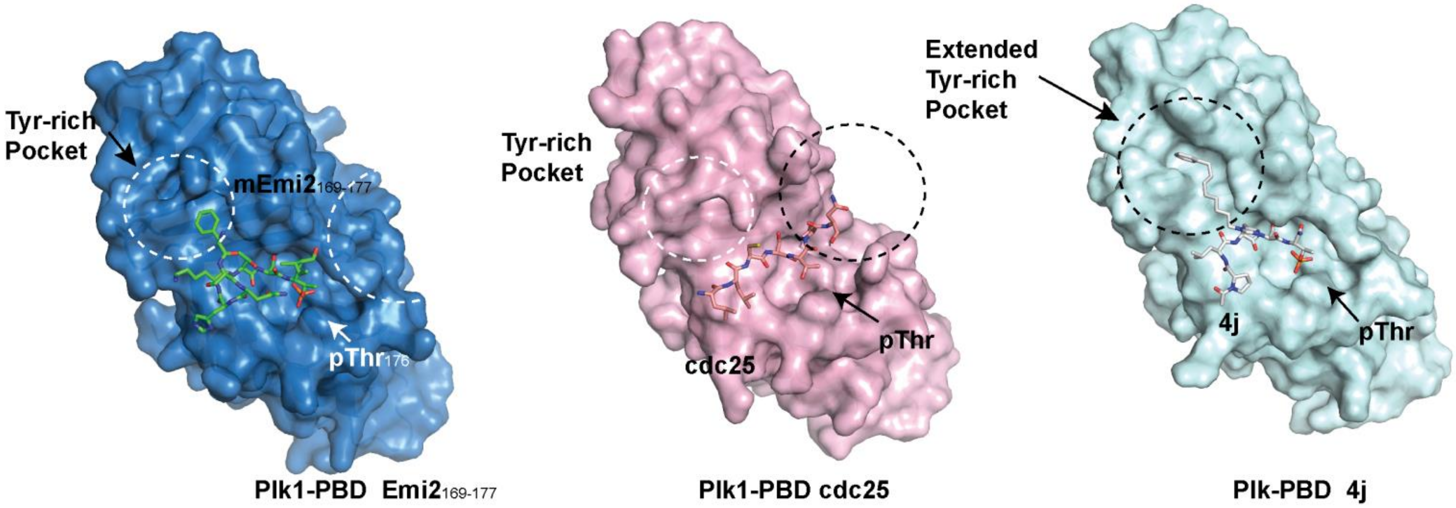

Figure S1. Conformational changes in the Plk1-PBD surface depending on the binding of different ligands. (Left) Structure of the complex Plk1-PBD·Emi2$^{169–177}$ (this study). Note that the Tyr-rich pocket was adjusted to accommodate the binding of Phe^{169} of Emi2. (Middle) Structure of the complex Plk1-PBD·cdc25 (Protein DataBank [PDB] ID: 2OJS) (Garcia-Alvarez et al., 2007). Note the similar conformations in Tyr-rich pockets; however, the C-terminal region after phosphor-Threonine induces changes in the Polo box domain (PBD) surface. (Right) Structure of the complex Plk1-PBD·4j (PDB: 3RQ7) (Liu et al., 2011). Note that the Tyr-rich pocket was extended by interaction of a long-chain alkyl phenyl group with a histidine.

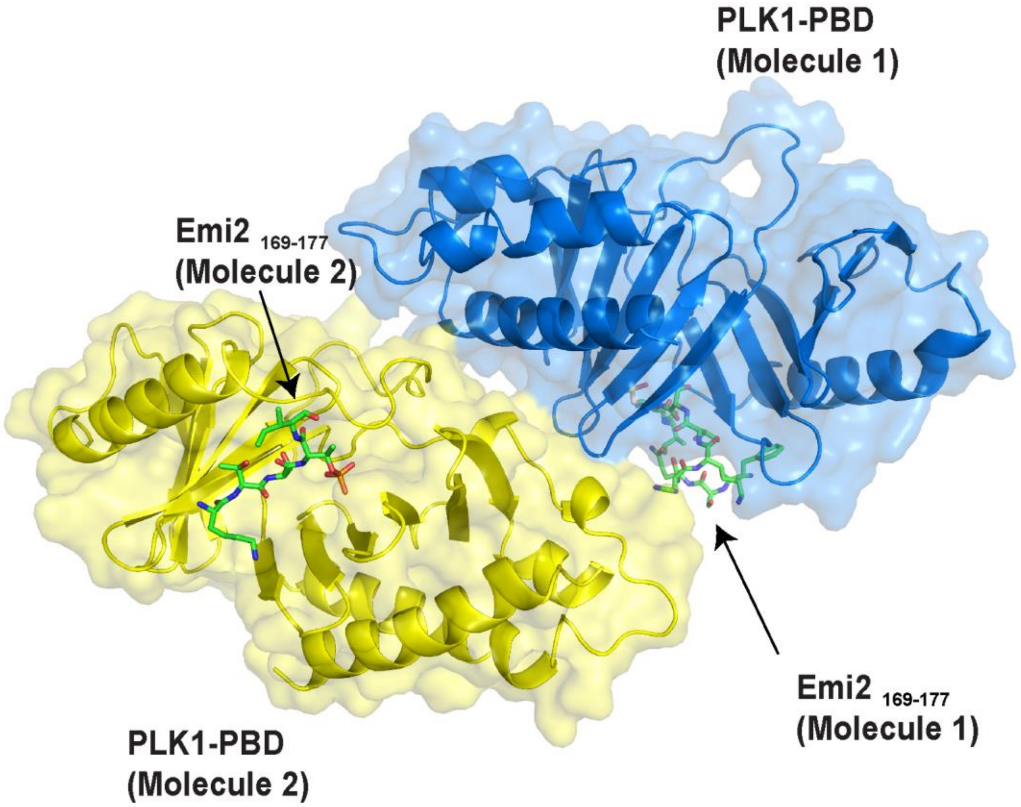

Figure S2. Structure of Plk1-PBD·Emi2$^{169–177}$ when shown in orientation different from that in Figure 2B. Although all amino acid residues in Emi2$^{169–177}$ bound to molecule 1 (Blue) of Plk1 Polo box domain (PBD) can be traced, only five amino acid residues (^{173}KTSpTI^{177}, where pT indicates phosphothreonine) of the Emi2 peptide have visible electron density when bound to molecule 2 (Yellow) of Plk1-PBD.

Supplementary Movie 1. Structure of Plk1-PBD· Emi2$^{146–177}$ and Emi2$^{169–177.}$ Plk1-PBD is presented as blue, while Emi2$^{146–177}$ or Emi2$^{169–177}$ were represented as green stick models. Electrostatic surface was represented as blue, positively charged to red, negatively charged. Conservation in PBD was mapped on surface by residue conservation (conservation decreases from blue to red as indicated by the bottom bar).
